# *Tritrichomonas foetus* infection, a cause of chronic diarrhea in the domestic cat

**DOI:** 10.1186/s13567-015-0169-0

**Published:** 2015-03-19

**Authors:** Chaoqun Yao, Liza S Köster

**Affiliations:** Department of Biomedical Sciences, Ross University School of Veterinary Medicine, P.O. Box 334, Basseterre, St. Kitts West Indies; Department of Clinical Sciences, Ross University School of Veterinary Medicine, P.O. Box 334, Basseterre, St. Kitts West Indies; One Health Center for Zoonoses and Tropical Veterinary Medicine, Ross University School of Veterinary Medicine, P.O. Box 334, Basseterre, St. Kitts West Indies

## Abstract

*Tritrichomonas foetus* is a very intriguing trichomonad protozoan with respect to its varied choice of residence in the different host species. It is an obligate parasite of the reproductive and the gastrointestinal tract of bovine and feline host respectively, leading to trichomonosis. Bovine trichomonosis is a sexually transmitted disease whereas feline trichomonosis is a disease with a purported fecal-oral route of spread. Further, the trichomonad is a commensal in the nasal passages, stomach, cecum and colon of swine host. Advances have been exponential in understanding the trichomonad biology and specifically feline trichomonosis since late 1990s and early 2000s when *T. foetus* was soundly determined to be a causative agent of chronic diarrhea in the domestic cat. It is a challenging task, even for a skilled investigator not to mention the busy clinical veterinarian, to keep up with the vast volume of information. Here we comprehensively reviewed the trichomonad biology, clinical manifestations, pathogenesis, host immunity, world map of distribution, risk factors, diagnosis and treatment. Risk factors associated with *T. foetus*-positive status in the domestic cat include young age, purebred, history of diarrhea, co-infections with other enteral pathogens. In addition, molecular similarity of bovine and feline isolates of *T. foetus* in DNA sequence was concisely discussed. The data presented serve as an information source for veterinarians, and investigators who are interested in biology of *T. foetus* and feline trichomonosis.

## Table of contents

1. Introduction
2. Molecular studies
3. Survival of trophozoites in the environment and possible transmission route
4. Clinical signs associated with gastrointestinal tract infection
5. Infection in the urogenital tract
6. Pathogenesis
7. Host immunity
8. Epidemiology
  8.1. Geographic distribution
  8.2. Risk factors
    8.2.1. Age
    8.2.2. Breed
    8.2.3. History of diarrhea
    8.2.4. Co-infection with other enteric protozoa
    8.2.5. Others
9. Diagnosis
10. Treatment
11. Conclusions
12. List of abbreviations
13. Competing interests
14. Authors’ contributions
15. Authors’ information
16. Acknowledgments
17. References

## 1. Introduction

**Figure 1:**
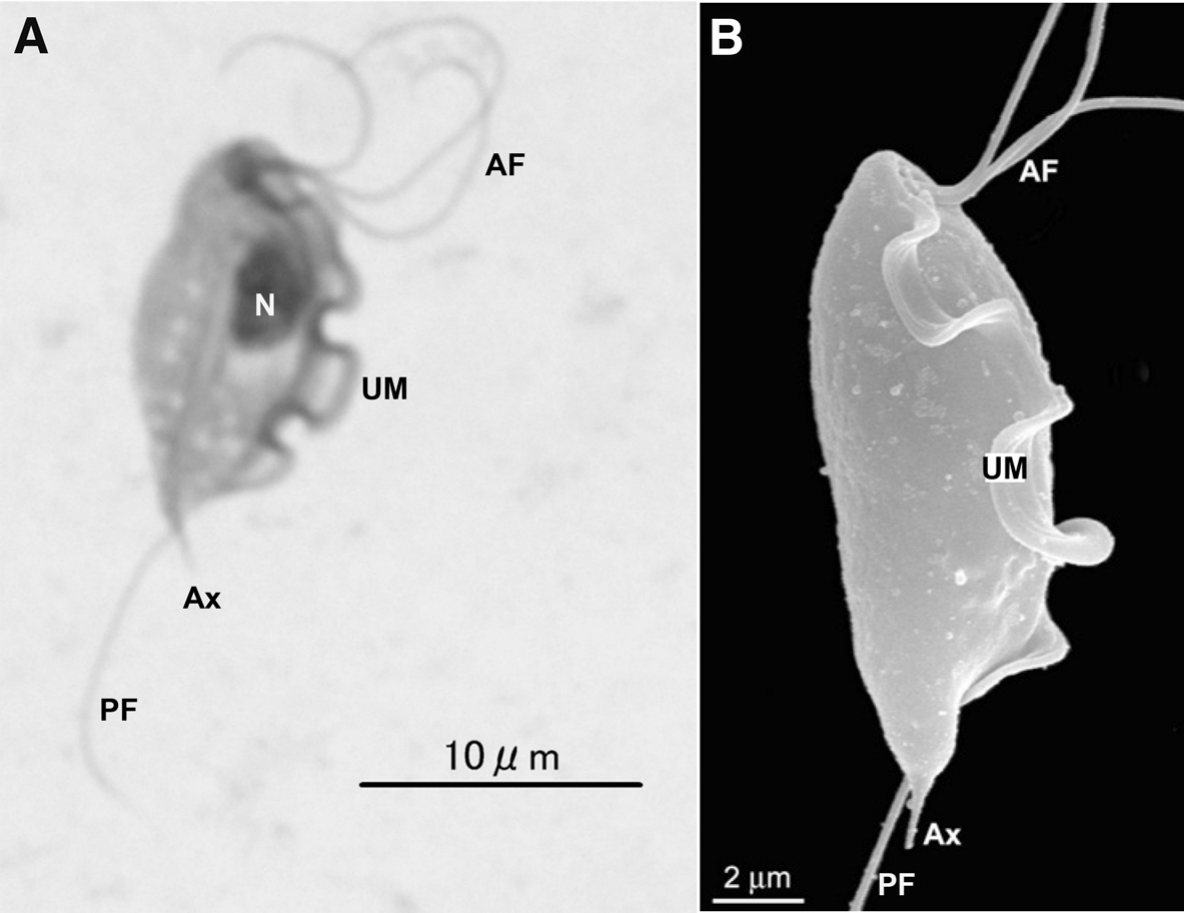
***Tritrichomonas foetus*** **trophozoites. A**: Giemsa-stain. **B**: scanning electron microscopy. AF: anterior flagella; Ax: axostyle; N: nucleus; PF: posterior flagellum; UM: undulating membrane. A and B: reproduced from Figure 1 of Doi et al. ([80]) and Figure 1b of Midlej et al. [81], respectively, with permission.

The genus *Tritrichomonas* belongs to the family Trichomonadidae. Among a few species of veterinary importance in the genus is *Tritrichomonas foetus. Tritrichomonas foetus* is fascinating both biologically and in its clinical manifestations in addition to being occasionally diagnosed in immunocompromised human beings [1]. It resides in the urogenital tract of cattle and causes bovine trichomonosis, a sexually transmitted disease with no approved treatment, throughout many geographic regions worldwide (bovine isolate) [2]. The same trichomonad species was soundly confirmed in 2003 to be the causative agent of chronic diarrhea in the domestic cat (feline isolate) [3,4] although the discovery of protozoan in these animals was made decades ago, as early as in 1928 [5]. Similar to other trichomonads such as human parasite *Trichomonas vaginalis*, *T. foetus* has only trophozoite stage although a pseudocyst stage is described [6-9]. Trophozoites reproduce asexually by longitudinal binary fission; no sexual reproduction has been ever discovered. They are pear- or spindle-shaped with three anterior flagella and one posterior flagellum. An undulating membrane extends along the whole length of the body and emerges as the posterior flagellum. The axostyle extends to the length of the cell and usually projects posteriorly. The size approximates 10-25 μm in length and 3-15 μm in width (Figure 1).

*Tritrichomonas foetus* has been a topic of review, especially on the clinical aspects of feline trichomonosis [10-15]. The major purpose of the current manuscript is to comprehensively review trichomonad biology, geographic distributions with compiling a world map of distribution, risk factors, host immunity and pathogenesis of the feline isolate. Of course it would not be complete without briefly going over clinical signs, diagnosis, treatment and prognosis of feline trichomonosis.

## 2. Molecular studies

Feline isolates found in the gastrointestinal (GI) tract of the domestic cat and bovine isolates in the urogenital tract of cattle are morphologically indistinguishable. An identity of 100% has been found within each of both feline and bovine isolates among more than 5000 base pair (bp) at 11 loci by DNA sequencing [16-19]. Nevertheless, there are minor, but consistent differences among these loci, ranging from 0.3% of internal transcribed spacer (ITS) and cysteine protease (CP) 7 and 8 to 1.9% of CP6 gene [16-19] (see Table 1 for details of each locus). Lately, 0.7% difference were found among eight protease inhibitors covering a total of 4674 bp, with individual sequence length ranging from 297 to 1145 bp by a transcriptomical approach. Further, CP8 and CP7 were the most transcribed gene in bovine and feline isolate, respectively [20]. Based on the molecular sequence differences along with disparity of experimental cross-infections and divergence in pathogenicity Walden et al. proposed a new name for the feline isolate. They named it *T. blagburni*, a new trichomonad species [21]. However, these authors did not show a clear separation of feline from bovine isolate, a requirement for naming a new species. The new nomenclature, if holding up, still waits to be accepted, which may take a while, especially among veterinarians. Nevertheless, comparative transcriptomics revealed near identical functional category distribution of expressed genes with no indication of molecular level divergence, which strongly suggested feline and bovine isolates were taxonomically two isolates of one species [20]. It is not our intension, nor the scope of this manuscript deals with such a debate on taxonomical status of the pathogen causing chronic diarrhea in the domestic cat. Consequently, the name of *T. foetus* is kept in the current review as well as in the title referring the feline isolate unless otherwise stated in order to be consistent with literature and to avoid confusion among readers.

**Table 1:** **Genetic similarity in percentage (%) between feline isolates and bovine isolates of** ***Tritrichomonas foetus***

	Bovine isolates*														**Refs**
**Feline isolates** ^**#**^	**ITS (2)**	**ITS-2 (10)**	**EF-1α (10)**	**CP8 (2)**	**CP1 (8)**	**CP2 (8)**	**CP4 (8)**	**CP5 (8)**	**CP6 (8)**	**CP7 (8)**	**CP8 (8)**	**CP9 (8)**	**MDH1 (8)**	**ITS1 + 2 (8)**	
ITS (4, 327)	99.7														[17]
ITS-2 (4, 368)		99.7													[16]
EF-1α (4, 783)			99.4												[16]
CP8 (4, 663)				99.7											[19]
CP1 (7, 503)					99.4										[18]
CP2 (7, 669)						96.7									[18]
CP4 (7, 273)							99.3								[18]
CP5 (7, 361)								99.2							[18]
CP6 (7, 318)									98.1						[18]
CP7 (7, 373)										99.7					[18]
CP8 (7, 907)											99.4				[18]
CP9 (7, 289)												99.3			[18]
MDH1 (7, 562)													99.6		[18]
ITS1 + 2 (7, 297)														99.7	[18]

CP, cysteine protease; MDH, malate dehydrogenase; ITS, internal transcribed spacer; EL, elongation factor.

*number of cattle isolates.

^#^first number - number of cat isolates; second number - length of sequence in base pair (bp).

In addition it is worth briefly describing another trichomonad, *T. suis*. The latter localizes at the nasal passages, stomach, cecum and colon of the domestic pig, and is morphologically indistinguishable from *T. foetus*. No differences have been found between *T. suis* and *T. foetus* bovine isolates at the molecular level such as random amplified polymorphic DNA (RAPD) [22-24], restriction fragment length polymorphism (RFLP) [24], and ITS-1 - 5.8S-rRNA - ITS-2 sequences [25,26]. Consequently they have been suggested synonyms, i.e. the same species [24,27]. Further sequence analysis of more than 5000 bp at 11 loci reveals 100% identity in all loci except elongation factor (EL)-1α and CP8 with 99.4% and 99.7%, respectively (Table 2). Together these molecular data confirm that *T. suis* and *T. foetus* bovine isolate are the same species, which makes *T. foetus* even more fascinating.

**Table 2:** **Genetic similarity in percentage (%) between** ***Tritrichomonas suis*** **and bovine isolates of** ***T. foetus***

	**Bovine isolates of** ***T. foetus*** *													**Refs.**
***T. suis*** ^**#**^	**ITS-2 (10)**	**EF-1α (10)**	**CP8 (2)**	**CP1 (8)**	**CP2 (8)**	**CP4 (8)**	**CP5 (8)**	**CP6 (8)**	**CP7 (8)**	**CP8 (8)**	**CP9 (8)**	**MDH1 (8)**	**ITS1 + 2 (8)**	
ITS-2 (4, 368)	100.0													[16]
EF-1α (4, 783)		99.4												[16]
CP8 (4, 663)			99.7											[19]
CP1 (4, 503)				100.0										[18]
CP2 (4, 669)					100.0									[18]
CP4 (4, 273)						100.0								[18]
CP5 (4, 361)							100.0							[18]
CP6 (4, 318)								100.0						[18]
CP7 (4, 373)									100.0					[18]
CP8 (4, 907)										100.0 (3); 99.9 (1)				[18]
CP9 (4, 289)											100.0			[18]
MDH1 (4, 562)												100.0		[18]
ITS1 + 2 (4, 297)													100.0	[18]

CP, cysteine protease; MDH, malate dehydrogenase; ITS, internal transcribed spacer.

*number of cattle isolates.

^#^first number - number of cat isolates; second number - length of sequence in base pair (bp).

## 3. Survival of trophozoites in the environment and possible transmission route

*Tritrichomonas foetus* like many other trichomonad protozoa has only trophozoites stage. Under the experimental conditions four pathogen-free and four *Cryptosporidium* sp. infected cats were inoculated via orogastric intubation with 2 × 10^6^ axenic *T. foetus*. All cats were persistently carrying the protozoan for the entire study period of over 200 days and experienced diarrhea that was self-resolved in seven weeks [28]. It is plausible that trophozoites are transmitted by a fecal-oral route from an infected cat to an uninfected one. By doing so trophozoites have to overcome challenges they face and survive in: 1) the environment they encounter during the period between being discharged from one host and being ingested by the next; and 2) the hostile gastric niche of the new host after ingestion and before moving down to the intestine. A few studies have been done on these areas.

In an experiment that normally formed cat feces that were first mixed with saline in a 1:1 ratio, resulting in no form, loose, puddles or piles, were spiked with 10-fold serially diluted trophozoites (2 × 10^2^ – 2 × 10^5^/gram feces) and stored at room temperature (23-25 °C) for various length periods of time. An accumulative rate of more than 80% of positive culture for feces stored for 6 and 24 h was obtained in both InPouch™ and in Modified Diamond’s Medium (MDM) [29]. It would be more relevant to find out how long the organisms would survive in diarrheic feces, which is currently unavailable. In another experiment cat food was spiked, the pathogens were cultivable for five consecutive days when MDM was used [30]. Similarly *T. foetus* trophozoites survived more than 3 h in feline urine and sauced cat food, 2 h on ground cat food although only half an hour in tap or distilled water [31]. These data collectively suggest that transmission is not solely limited to close contact between cats. Contamination of food and water by the trichomonads, although less likely in the latter, may be an important route for transmission. Further, garden slugs common in Sydney, Australia were fed cat foods spiked with 10^6^ *T. foetus* feline isolate trophozoites per gram. One hundred percent and 83% of the Leopard slugs and the Yellow cellar slugs shed viable *T. foetus* in their feces, respectively, which was cultivable in MDM. Therefore slugs may facilitate the transmission of *T. foetus* among cats [30] as a vector. It is also plausible that these slugs may serve as transport hosts upon accidental ingestion by cats, which needs to be confirmed.

## 4. Clinical signs associated with gastrointestinal tract infection

Gastrointestinal disease has been demonstrated in cats experimentally infected with *T. foetus*, an organism not considered part of the normal feline microbiome [3,28,32]. It is possible to isolate *T. foetus* from a healthy cat as subclinical shedding does occur [33]. Infection can be demonstrated as early as 2 to 7 days after orogastric inoculation [28]. In experimental infection, *T. foetus* is limited to the ileum, cecum and colon [28]. Clinical signs vary from subclinical to intractable large bowel diarrhea [3]. Typical clinical signs in natural infections are chronic or intermittent large bowel diarrhea reported in about 61% to 64% of infected cats, with many cats having no reported diarrhea in the 6 months preceding diagnosis [34,35]. The feces has been described as yellow-green in color and malodorous with typical signs of colitis including fresh blood, mucous, fecal incontinence, tenesmus and flatulence [3,36]. The consistency of the feces has been described most commonly as semi-formed to cow pat [28]. An objective fecal scoring system has been used in studies: a scale of 1 to 5 based on consistency, with a score of 1 representing watery feces and a score of 5 is dry and firm feces. Trichomonad infected cats fecal score have been described to range from 3 to 5 [33]. Up to as many as 20% of infected cats have reported systemic signs including anorexia, depression, vomiting and weight loss with a case report describing a kitten as pyrexic and experimental infections reporting vomiting and fever [34,36,37]. In a study examining experimental infection (*n* = 8), only two cats demonstrated clinical signs of disease, fecal blood and mucous at nine days post-infection (dpi), and fever with vomiting 21 dpi [32]. Interestingly, motile trichomonads were observed in intestinal content culture in only three of the eight cats infected, none of which showed clinical signs. Mortality is extremely rare and only reported in kittens. The first report of mortality was in the study that first described natural and experimental trichomonosis in kittens in 1928 with a feline isolate [5]. All nine kittens diagnosed with naturally acquired infection, and five of the six experimentally infected kittens, wasted and died within five to ten days and four to nine days of diagnosis respectively [5]. The second report that documents mortality was a prevalence study examining *T. foetus* infection in cats with diarrhea in a shelter colony in Italy [38]. One 7-month old kitten housed in this shelter with confirmed *T. foetus* infection succumbed to suspected septic shock despite having started ronidazole therapy two days prior [38].

Clinical signs are reported to persist for 5 to 24 months (median 9 months) from the time of diagnosis [33]. More than half of the cats that go into clinical remission will have PCR evidence of trichomonad infection (asymptomatic carrier), and many of these cats will relapse for a short duration, often with worse diarrhea [33]. Not surprisingly, the number of other cats in the same household will negatively impact the time from diagnosis to resolution of clinical signs [33]. Infection with *T. foetus* is not unlikely in single-cat households even in cats that have lived in isolation for years, as they can acquire the infection in the early stages of their lives [34]. Trichomonad infections can occur as co-infections, most notably *Giardia* species and coccidia [3,34,35,39]. More severe diarrhea has been reported in four cats experimentally infected with *T. foetus* and *Cryptosporidium* species, despite the latter organism having a tropism for the small intestines [28]. In surveys of naturally acquired trichomonad infections the severity of diarrhea has not reported to be worsened by co-infection with enteric parasites [35].

## 5. Infection in the urogenital tract

In addition to the GI tract *T. foetus* is also found in the urogenital tract of the domestic cat at least once. Dahlgren et al. reported the first case of *T. foetus* in the uterus of a cat with pyometra [40]. In this case wet mounts made directly from the fluid collected from the surgically removed uterus revealed microscopically numerous motile flagellated protozoa. They were 13–17 × 5–7 μm in size with undulating membrane, suggesting *T. foetus*. Further, PCR detected an expected size band product of 343 bp in length using primers PRF3 and PRF4. The PCR product was sequenced and the sequence was 100% identical to *T. foetus* feline isolate sequences, which confirmed the identity of the protozoan causing feline pyometra as *T. foetus* [40]. *T. foetus* was also found in the feces of three other cats in the same household, one of which had chronic diarrhea [40]. These authors were unable to conclude that the infection was sexually transmitted even though the female mated repeatedly with *T. foetus*-positive male with chronic diarrhea [40].

In a comprehensive study Gray et al. used parallel samples collected in USA from both the urogenital tract as a result of ovariohysterectomy or castration and feces of purebred cats [41]. Direct microscopy, Immunohistochemical analysis and PCR were used to test each sample for the presence of *T. foetus* among the two sets of samples. *Tritrichomonas foetus* infections were detected by PCR in 25% (15/61) cats and 67% catteries (22/33). Nevertheless, *T. foetus* was never detected in the urogenital tract of any cats, including the 15 *T. foetus*-positive cats in feces. These authors concluded that no evidence of urogenital tract colonization by *T. foetus* was detected and that urogenital tract infection with *T. foetus* very unlikely plays an important role in overall disease transmission [41]. Collectively this data demonstrates that infection of the urogenital tract by *T. foetus* does occur and may cause pyometra, but it is a rare event especially among the healthy cats presenting to clinics for ovariohysterectomy or castration.

## 6. Pathogenesis

**Figure 2:**
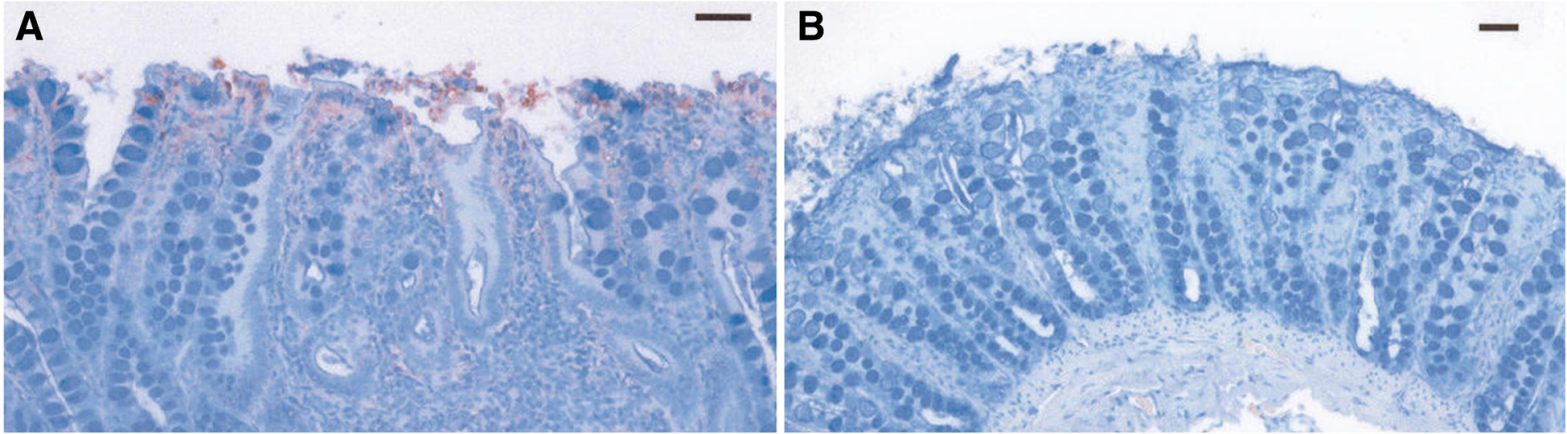
***Tritrichomonas foetus*** **antigens in colonic mucosal biopsy specimens.** Immunolabeled trichomonads (red stained organisms) in panel **A** are located on surface enterocytes and within superficial mucus and detritus of the cecal mucosa. Panel **B** is a negative control omitting primary antibody. Bar = 100 μm. Reproduced with permission [28].

In the above mentioned experiment, four pathogen-free and four *Cryptosporidium* sp. infected cats were inoculated with axenic *T. foetus*. All cats experienced self-resolved diarrhea for weeks. *Tritrichomonas foetus* was isolated from the ileum, cecum, and colon during necropsy. It was further shown that protozoa and its surface-located antigen were detected on surface epithelia and within superficial detritus of the cecal and colonic mucosa (Figure 2) [28]. Similarly in naturally infected cats the parasites were generally present in close proximity to the mucosal surface and less frequently in the lumen of colonic crypts. Mild-to-moderate lymphoplasmacytic and neutrophilic colitis, crypt epithelial cell hypertrophy, hyperplasia and increased mitotic activity, loss of goblet cells were associated with the presence of trichomonads in colon [42]. Consequently in both experimental and natural infections the predilection sites for *T. foetus* trophozoites are the epithelial surface and crypts of cecum and colon.

Burgess et al. found that *T. foetus* bovine isolate was highly cytotoxic to Hela cells, a human cervical cell line, and early bovine lymphosarcoma (BL-3) but displayed low levels of cytotoxicity against Vero cells [43]. The latter are African green monkey kidney cells [43]. This isolate also adheres to bovine vaginal epithelial cells (BVECs) in an in vitro assay. The adhesion process was first initiated by the posterior flagellum followed by the body. IgG_1_ antibodies inhibited adherence, whereas IgG_2_ did not [44]. Furthermore, purified lipophosphoglycan (LPG) of *T. foetus* bovine isolate inhibited the binding of *T. foetus* to BVECs by competing with the protozoa in specific receptor – ligand interactions [45]. Adhesion of *T. foetus* resulted in extensive damage of BVEC monolayers [45]. This cytopathic effect was the results of apoptosis induced by *T. foetus*. A painstaking research pinpointed that CP8 of 30 kDa was the major player in *T. foetus* inducing BVEC apoptosis [46]. Collectively the data from multiple research groups demonstrate that *T. foetus* bovine isolate causes cell death of BVECs by means of apoptosis, in which CPs such as CP8 plays a pivotal role. It is not clear whether the same occurs in vivo in infected cows and heifers, which waits to be confirmed.

**Figure 3:**
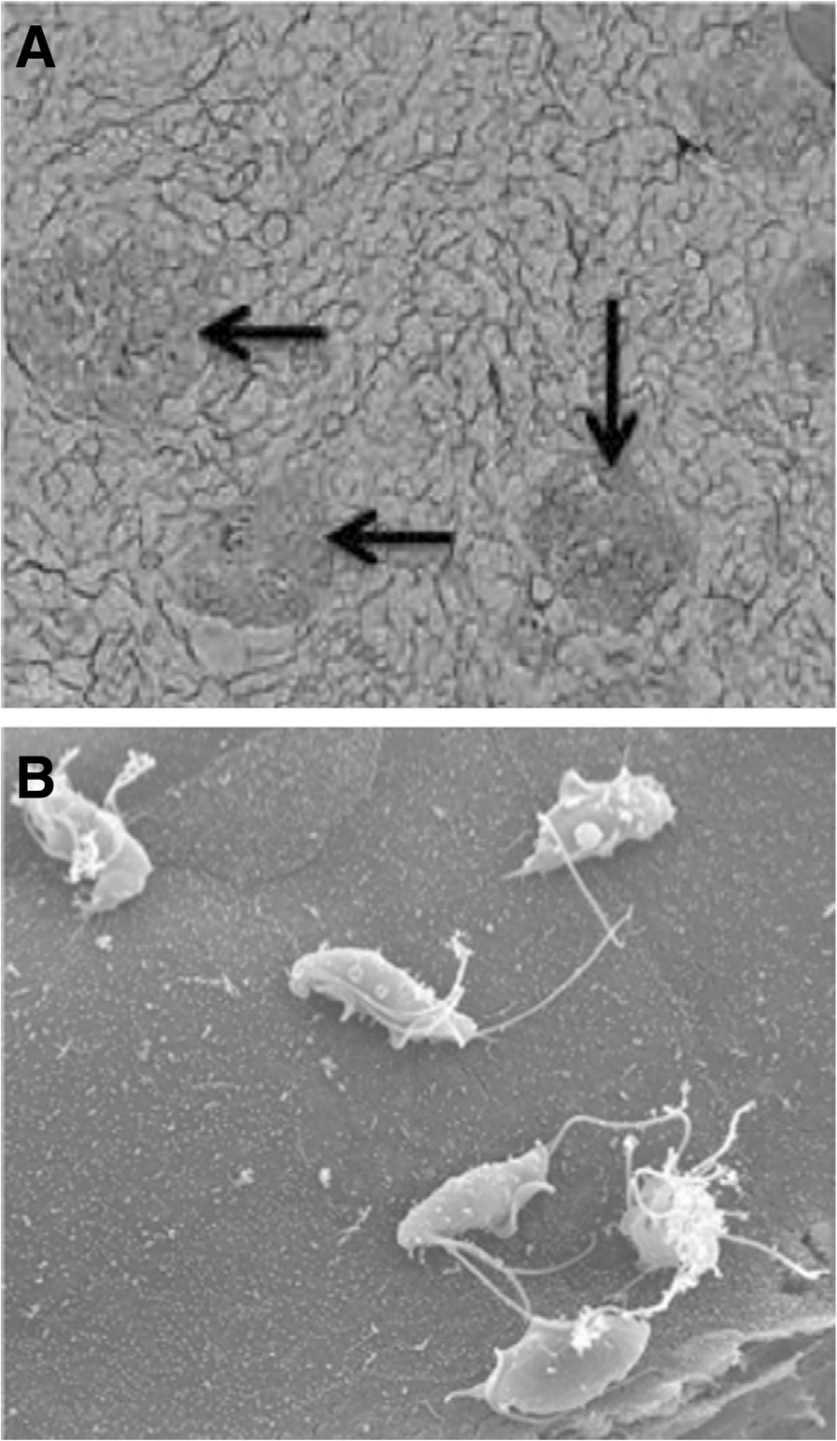
**Scanning electron microscopy of** ***Tritrichomonas foetus*** **adhesion to porcine intestinal epithelial cell (IPEC)-J2 monolayers. A**. Aggregates of trophozoites adhering to IPEC-J2 monolayers (arrows). **B**. Six trophoziotes adhering to a single IPEC-J2 cell. Reproduced with permission [47].

In an in vitro analysis Tolbert et al. demonstrated that feline *T. foetus* trophozoites adhere to monolayers of the porcine intestinal epithelial cell line (IPEC)-J2 cells [47] (Figure 3). The reason for using a porcine jejunal cell line was the unavailability of epithelial cell line of feline origin. It is worthy of noting that *T. suis*, a synonym of *T. foetus* resides in the GI tract of pigs without causing clinical signs as mentioned in the section of molecular studies. So IPEC-J2 may represent the second best in vitro model. These authors showed that adhesion was via specific receptor – ligand interaction, required viable trichomonad cells and was independent of cytoskeletal activity of the trophozoites [47]. Lately the same authors elegantly demonstrated that feline *T. foetus* trophozoites progressively destroyed IPEC-J2 cell monolayer via apoptosis. The protozoan promoted a direct contact – dependent activation of intestinal epithelial cell apoptosis signaling. This pathologic effect depended upon *T. foetus* – cell associated cysteine proteases [48]. Effect of bovine *T. foetus* on epithelial cultured cells was investigated using clonal populations of the protozoan. Five clonal populations destroyed epithelial monolayers at different degrees, ranging from 25% to 55% despite similar cytoadhesion levels and whole-cell protease activity. They also showed various degrees of contact – dependent and contact – independent cytotoxicity. Contact – independent cytotoxicity was strictly related to the degree of enzyme activation of an extracellular protease [49]. Furthermore, it was shown that extracellular proteinases such as CP8 of *T. foetus* bovine isolate cleaved C3b (the bigger fragment of two C3 component in the original paper designed as C3a) to small fragments, which could play a role in evasion of complement killing in vivo as a result of preventing completion of complement activation cascade [50].

Taken together, the pathogenesis of *T. foetus* on the intestinal epithelial cells is both contact – dependent and contact – independent. In the former, cytopathic effect is mainly via apoptosis induced by cell-associated proteases, whereas extracellular proteases are the major players in contact – independent cytotoxicity. Extracellular proteases may also play a role in evading complement killing. Consequently proteases are a major player although other uncharacterized molecules may also contribute to pathogenesis of this intriguing trichomonad. Consistent with the notion, 483 and 445 bovine and feline isolates of *T. foetus* transcripts were determined to be proteases by a transcriptomical approach. Among these 389 and 346 were CP, respectively [20].

## 7. Host immunity

There is significant paucity of literature on immunity of feline hosts to microbial infections in general and to *T. foetus* infections in the GI tract in specific. The latter is understandable provided that the trichomonad has been ascribed to be causative agents only in last 15 or so years. In general IgA is a pivotal player in mucosal secretion of cat’s GI tract [51,52]. Consequently IgA profile in the GI tract among different age groups of various breeds is crucial to understand host immunity against and susceptibility to microbial infections. Unfortunately this information is very scarce at present. It was found very low concentrations of hyperimmune serum (up to 1:640 dilution, the highest available title in the experiments) promoted significant enhancement of killing of *T. foetus* bovine isolate by the alternative pathway of bovine complement [53]. Nevertheless, extracellular proteinases such as CP8 of *T. foetus* bovine isolate cleaved C3b to small inactive fragments, which may counteract this host defense system [50].

## 8. Epidemiology

### 8.1. Geographic distribution

Although *T. foetus* was reported in nine kittens with diarrhea as early as 1928 [5] its status as an etiological pathogen of chronic diarrhea of the domestic cat was only confirmed very recently. Gookin et al. established an association between diarrhea and *T. foetus* infections in the domestic cat in the USA [3]. They further determined that this trichomonad was the etiological agent of feline trichomonal diarrhea using rRNA gene sequence analysis; restriction enzyme digest mapping; and light, transmission, and scanning electron microscopy [4].

**Figure 4:**
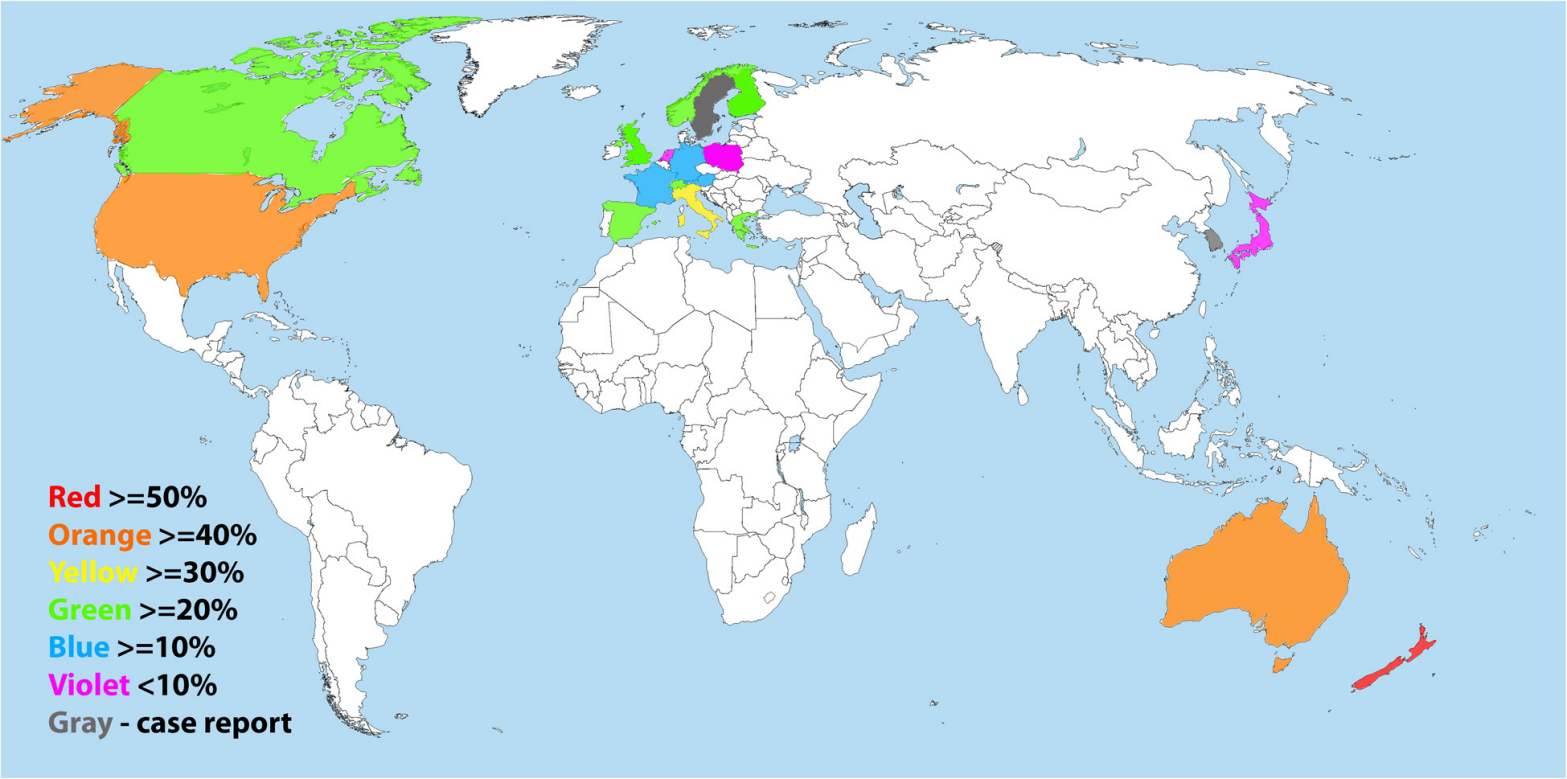
**Geographical distribution of surveys for and case report of** ***Tritrichomonas foetus*** **detected positive cats worldwide.** The prevalence of *T. foetus* infection of each regional study is reported although is not representative of the true prevalence of the entire country. Highest rate is used in countries with more than one regional report on prevalence.

*Tritrichomonas foetus* has been diagnosed in the domestic cat in many geographic regions. To the best of our knowledge its geographic distribution has covered four continents including Europe (Austria, Finland, France, Germany, Greece, Italy, Netherland, Norway, Poland, Spain, Sweden, Switzerland, and UK), North America (Canada and USA), Australia/Oceania (Australia and New Zealand), and Asia (Japan and South Korea) (Figure 4). In some regions only case reports were available, whereas in others survey data were generated (Table 3). Many of the survey data were collected from diarrheic cats, show cat, cats in catteries, or cats presenting to veterinarians and veterinary clinics. Consequently there is a bias associated with such samples, i.e., a bias resulting in higher positive rate than cross-sectional samples that would have been collected from pet owners under the most scenarios. For instance in New Zealand, 22 samples of show cats revealed a positive rate of 82%. In USA diarrheic cats had positive rate as low as 6% in one study [54], and as high as 40% in another [55]. Figure 4 shows the different geographic regions of the world where surveys have been conducted and/or cases have been reported.

**Table 3:** **Cases and prevalence of feline trichomonosis throughout the world**

**Country**	**Year**	**Sources (cat examined)**	**Diagnosis**	**Case report**	**Prevalence (%)**	**Refs**
Australia		Vet Clinic	M;C;PCR;S	16		[63]
Australia	2006-2010	Vet Clinic	M: PCR	13		[82]
	2008-2009	Cattery (59)	PCR		42.4	
Australia	2006-2007	Cattery (82)	C;PCR		0	[83]
		Shelter (52)			0	
Austria & Germany		Fecal samples (31)	PCR,S		19.4	[84]
Austria	1997-2008	Necropsy (96); Organ samples (6)	chromogenic in situ hybridization; PCR; S		2.9	[71]
Canada		Cats with chronical diarrhea (1)	PCR	1		[85]
Canada	2011-2012	Cat Clinic (140)	C;PCR		0.7	[61]
		Cat show (55)			23.6	
		Humane Society (46)			0	
European Union – 15 countries	2009-2010	Diarrheic cats (1840)	PCR,S		9.0	[59,86]
Finland	2008-2010	Diagnostic samples (60)	PCR		28.3	[87]
France	2009-2010	Cat show (140)	C;S		14.3	[58]
Germany		Diarrheic cats (3)	M, PCR, S	3		[88]
Germany	2008	Cat shows (230)	C;PCR;S		15.7	[35]
Greece		Fecal samples (30)	PCR		20.0	[89]
Italy	2008-2010	Pet (181), sheltered cats (54), stray cats (28)	C		0	[90]
Italy		Healthy pets (146)	PCR		2.1	[65]
Italy	2006	Rescued Cats (74)	M;C;PCR		32.4	[38]
Japan	2008	Animal Hospitals (147)	C;PCR;S		8.8	[80]
Netherland	2006	Diarrheic cats (53)	PCR		2	[91]
		Cattery (47)			4	
		pet (54)			0	
New Zealand	2006	Cat shows (22)	C;PCR		81.8	[64]
Norway	2006	Vet Clinic	M; PCR;S	3		[40]
Norway	2009	Cat show (52)	C; PCR;S		21.2	[62]
Poland		Vet Clinics (135)	C;PCR		7.3	[92]
South Korea	2008	Vet Clinic	M;C;PCR	2		[93]
Spain		Cattery (20)	M;C;PCR		25.0	[94]
Sweden		Diarrheic cats (1)		1		[95]
Switzerland	2007	Diarrheic cats (45)	C;PCR;S		24.4	[96]
Switzerland	2007-2008	Vet Clinic (105)	C;PCR		25.7	[97]
UK	2004	Diarrheic cats (1)	M; PCR	1		[36]
UK	2006	Diarrheic cats (111)	PCR		14.4	[57]
UK	2010 - 2012	Diarrheic cats (1882)	PCR		18.8	[66]
UK		Vet Clinic (163)	PCR		20.0	[98]
USA		Diarrheic cats (26)	M;C;PCR	26		[33]
USA		Diarrheic cats (32)	M;C	32		[3]
USA		Diarrheic cats (1)	M;C;PCR	1		[75]
USA		Diarrheic cats (4)	C; PCR	4		[74]
USA		Diarrheic cats (4)	C; PCR	4		[78]
USA	2006-2009	Vet Clinic (104)	PCR	104		[34]
USA		Diarrheic cat (7)	M;C;PCR	7		[42]
USA		nondiarrheic cats (53)	C;PCR		0.0	[54]
		Diarrheic cats (170)			5.9	
USA		Fecal samples (173)	C;PCR		9.8	[39]
USA		Catteries (61)	M; PCR		24.6	[41]
USA	2001	Cat show (117)	M;C;PCR		30.8	[60]
USA	2009-2012	Diarrheic cats (68)	PCR		39.7	[55]

C: Culture; M: Microscopy; PCR: polymerase chain reaction; S: Sequencing.

### 8.2. Risk factors

Odds ratio (OR) is commonly used to quantitatively gauge risk factors. An OR is a measure of association between an exposure and an outcome [56]. In this section OR will be used either directly taken from the original publications or calculated by us if data allow to do so. Risk factors discussed in the section include age and breed of the animals, a history of diarrhea, co-infection with other enteral protozoa and housing conditions etc.

#### 8.2.1. Age

An association between age of infected cats and *T. foetus* infections has been probed in many studies, of which several were showed in Table 4. A common notion is that cats of one year old or younger are more susceptible to *T. foetus*. However, data presented in Table 4 did not show a clear picture at all. OR for cats ≤ 1 year old generated from data collected from France and UK were 2.5 (*P* = 0.057) and 13.4 (*P* < 0.01), respectively [57,58]. Further, Galian et al. reported a positive association between age and *T. foetus* prevalence among 1391 fecal samples submitted to a diagnostic laboratory originated from 15 countries in the European Union [59]. They found 10.4%, 5.5%, 2.5%, 3.5% and 0% *T. foetus*-positive cats among age groups ≤1 year old, 2-7, 8-11, 12-15 and ≥15 years old, respectively [59]. However, data from Germany yielded a contradicted picture with younger cats marginally less susceptible with OR 0.9 (*P* < 0.05) [35]. Also no association was found from data collected from Canada, Norway, and USA [60-62] (Table 4). Collectively data from multiple countries do not consistently support a notion that *T. foetus* infections occur more often in the domestic cat of one year old or younger.

**Table 4:** **Risk factors related to feline infections by** ***Tritrichomonas foetus*** ^**¶**^

**Country**	**Age in month**	**Sex-male**	**Purebred**	**Housing**	**History of diarrhea in past 6 months**	**Food**	**Refs**
Canada	<6 – 0.6 (0.1–3.4)	2.0 (0.5–7.9)	26.3 (3.8–1142.2)**	>5 cats per house – 4.6 (1.3–20.8)**	Another cat – 4.4 (1.3–16.2)**	Fed a raw food diet – 5.4 (1.5–19.6)**	[61]
					Self – 1.7 (0.4–6.4)		
France	<12 – ^#^2.5(0.6-10.2)^+^	NC	NC	NC		NC	[58]
Germany	≤12 – 0.9 (0.7-1.0)*	NC	Norwegian Forest – 25.9 (7.6-87.7)***	NC	Another cat – 3.2 (1.2–9.9)**	NC	[35]
					Self – 3.2 (1.1- 8.7)*		
Norway	NC	NC		NC	NC (*P* = 0.1)		[62]
UK	6-12 (29.4%); >12 (15.2%)***		Pedigree (37.8%);				[66]
			Non-pedigree (6.0%)***				
UK	≤12, ^#^13.4(1.7-107.7)**	NC	^#^ > 999.9				[57]
			Siamese and Bengal ^#^7.3 (2.1-25.9)**				
USA	Infected mean 8 vs non- infected mean 64.8***		Abyssinians, Siamese and Bengal**				[54]
USA		^#^> 999.9**					[41]
USA	NC	NC			Self – 3.5 (1.1-11.3)*		[60]

^¶^Odds ratio is given; 95% confidence interval is in bracket. Blank cells: no data available.

^#^:Calculated by the authors of current manuscript according to [56]; *P* value from the original paper.

*P < 0.05; **P < 0.01; ***P < 0.001; ^+^ *P* = 0.057.

NC: No correlation was detected.

A metadata analysis on OR of cat of one year old or younger was performed by the current authors on available data in publications, which all happened to originate from various European countries (Table 5). The cats in this age group had an OR of 2.1 (1.7-2.6) than older animals. Therefore, young age is a risk factor for *T. foetus* infection.

**Table 5:** **Data included in metadata analysis of odds ratio of young cats with** ***Tritrichonomas foetus*** **infections**

**One year old or younger**			**Older than one year old**			**Reference**
**Positive**	**Negative**	**Total**	**Positive**	**Negative**	**Total**	
13	35	48	1	36	37	[57]
17	76	93	3	44	47	[58]
65	563	628	33	730	763	[59]
5	14	19	6	26	32	[62]
100	313	413	92	515	607	[66]
9	3	12	1	22	23	[96]
209	1004	1213	136	1373	1509	

#### 8.2.2. Breed

There are many different pure breed and crossbreeds of the domestic cat throughout the world. Some breeds are more popular than the others in certain geographic regions. Many veterinarians have observed that purebreds present more often to clinics than crossbreds due to *T. foetus* infections. Among six sets of data all except one found this is true (Table 4). The only exception might be due to a small sample size. In this case, 20 *T. foetus*-positive samples were found in 140 show cats [58]. Purebreds have a much higher OR to be *T. foetus* positive than non-purebreds, which range from 26.3 in Canada [61] to >999.9 in UK [57]. Further analysis showed that a few breeds with a higher OR, such as 7.3 for Siamese and Bengal in UK [57], and 25.9 for Norwegian Forest in Germany [35]. Abyssinians, Siamese and Bengal were found more likely to be *T. foetus* positive in a survey carried out in USA [54]. Collectively purebred is a risk factor for *T. foetus* infections in general, and Abyssinians, Siamese, Bengal and Norwegian Forest in specific. Nevertheless, it is worthy of mentioning that some studies sampled purebred cats from cat breeding centers with a densely housed population. Under this scenario increased incidence of infection might be due to the reliance on close and direct contact among animals.

#### 8.2.3. History of diarrhea

History of diarrhea can be the *T. foetus* positive cat itself, or the other cats sharing the same household in the past, say 6 months. In the former, two studies showed OR 3.2 (*P* < 0.05) in Germany [35] and 3.5 (*P* < 0.05) in USA [60]. Third study in Canada showed an OR 1.7 without statistical significance [61]. For the latter, two investigations resulted in ORs of 4.4 (*P* < 0.01) in Canada [61] and 3.2 in Germany (*P* < 0.01) [35]. Therefore, history of diarrhea in the past six months, no matter whether it is the presenting cat itself or its playmates, rendered a cat a threefold higher chance of having a *T. foetus*-positive status.

#### 8.2.4. Co-infection with other enteric protozoa

Enteral protozoa of the domestic cat include, but are not limited to, *T. foetus*, *Giardia duodenalis*, *Cryptosporidium* spp. *Toxoplasma gondii*, *Isospora* spp. and *Sarcocystis* spp. Co-infection of these is not unusual. For example, five of 16 *T. foetus* positive cats were *Giardia* sp. positive in Australia [63]; five of 18 *T. foetus* positive cats were *Giardia* sp. positive in New Zealand [64]; ten, six and one of 36 *T. foetus* positive cats were *Giardia* sp., *Isospora* sp., and *Cryptosporidium* sp. positive, respectively, three were co-infected by all except *Cryptosporidium* sp. in a survey carried out in Germany [35]. In a survey of 146 fecal samples in Italy, co-infections were not found although 15, 11, 3, and 3 were positive for *T. gondii*, *G. duodenalis*, *Cryptosporidium* sp. and *T. foetus*, respectively [65]. A detail analysis of enteropathogen co-infection in UK cats with diarrhea was carried out. There was greater co-occurrence than random of *G. duodenalis* with either *T. foetus* or *Cryptosporidium* sp. respectively. Further a greater three-way co-occurrence existed among these three enteral protozoa [66]. These data collectively show that these enteral protozoa render greater risk for the domestic cat to be infected with *T. foetus* even though they may locate at different niches than *T. foetus*.

#### 8.2.5. Others

A few other factors have been analyzed for and found their association with *T. foetus*-positive status occasionally. One is sex. Among seven studies only one demonstrated being a male kitten is a great risk factor (*P* < 0.01) [41]. Another one is housing with more than one other cat. One of four surveys showed sharing house with more than five cats had an OR 4.6 (*P* < 0.01) [61]. The third one is food, one of three investigations resulted in an OR 5.4 for feeding raw food diet (*P* < 0.01) [61]. What causes this disparity is beyond the scope of this manuscript. Plausible reasons include small sample sizes and confounding factors.

## 9. Diagnosis

*Tritrichomonas foetus* infection should be suspected in a cat with recent (<6 months) clinical signs of chronic large bowel diarrhea, with highest risk in densely housed young, purebred, show cats [3,60]. There are no differences in the signalment between *Giardia* sp. and *T. foetus* infected cats and co-infections are common enough (12%) to warrant testing for *T. foetus* despite the diagnosis of other enteric protozoal infection, although *Giardia* sp. is a small bowel parasite and the clinical signs should distinguish the infections. There have been no hematological or biochemical abnormalities reported in the literature. Diagnosis of a trichomonad infection is made by either the demonstration of the trophozoite on a saline diluted direct fecal smear (14.7% sensitivity), culture by inoculating MDM (26.4% sensitivity) or the commercially available InPouch™ TF medium (InPouch TF; BioMed Diagnostics, Inc, White City, OR USA) (58.8% sensitivity) or the extraction of DNA in feces and the amplification of *T. foetus* rDNA by the use of PCR [13,60]. Fecal samples include either a voided stool or collected by manual extraction with the aid of fecal loops or by a colon flush technique. The technique of colon flush is demonstrated in a video clip the North Carolina (NC) State University, College of Veterinary Medicine (CVM) website (Colon Flush Technique [67]). Fecal wet preparation is viewed under 40 × magnification. The motile characteristic of *T. foetus* differ from *Giardia* sp. in that it has a forward motility in contrast to a falling leaf motility of *Giardia* sp. as demonstrated in a video clip from the NC State University CVM website (*T. foetus* vs. *Giardia* [68]). Failing to distinguish the two trophozoites on microscopy, *Giardia* sp. can be confirmed by fecal enzyme-linked immunosorbent assay for *Giardia-*specific antigen [60]. In cases where *T. foetus* is suspected despite negative microscopy or fecal culture, when a faster turn-around time than culture is required, or for confirmation of an organism visible on microscopy, *T. foetus* cannot precisely be distinguished microscopically from *Pentatrichomonas hominis,* a commensal trichomonad, a commercially available PCR assay is available targeting part of the 18 s ribosomal RNA (rRNA). Submission of samples should be diarrheic and free from litter as formed stool rarely tests positive even if a subclinical shedder (NC State University CVM, Submission of Samples for *T. foetus* PCR Testing [69]). The sample, roughly the size of a lima bean should be submitted in a sterile tube and the remainder of the volume filled with isopropyl alcohol, without the need for refrigeration. Unfortunately PCR cannot prove the absence of infection.

Chronic experimentally induced infection with *T. foetus* is limited to the colon, cecum and ileum [28]. The distribution of *T. foetus* organisms within the colon is not homogenous and histopathological diagnosis on samples procured by necropsy, surgery or endoscopy in cats with naturally acquired infection had a sensitivity of 56% [42]. The probability of diagnosing *T. foetus* infection on histopathology is increased with the number of samples submitted. Thus examination of multiple samples will increase the likelihood of diagnosis with a minimum of 6 colon samples required to have a ≥95% confidence of detecting *T. foetus* on at least one sample [42]. A species (sequence) specific fluorescent antibody in situ hybridization (FISH) probe has been developed for the detection of *T. foetus* on formalin fixed samples allowing confirmation of location and molecular identification of a trichomonad [70]. A study examined an chromogenic in situ hybridization (CISH) technique using *T. foetus* and *P. hominis* probes on formalin archived small and large intestinal samples collected from young (4 weeks to 2 years of age) cats with diarrhea [71]. This technique is apparently more reliable than FISH technique as mammalian red blood cells, roughly the same size as trichomonads, auto-fluoresce. Four of the 102 samples were found to be positive, with three testing positive for the *T. foetus* probe and one with the *P. hominis* probe.

## 10. Treatment

The natural course of diarrhea in cats infected with *T. foetus* is waxing and waning, giving the false impression that therapy may be effective, but often relapsing after discontinuation of treatment [3]. Trichomonads rely on hydrogenosomal fermentation of pyruvate making them susceptibility to 5-nitroimidazole antibiotics, such as metronidazole, tinidazole and ronidazole [72]. Therapeutics reported in the literature include: paromomycin, fenbendazole, furazolidone, nitazoxanide, metronidazole, tinidazole and ronidazole [3,28]. One of the earliest reported descriptions of treatment was paromomycin, an aminoglycoside used to treat *Trichomonas vaginalis*, a human protozoal infection of the genital tract, at the same dose used to treat cryptosporidiosis in cats [3]. In the 25 cats studied, evaluated 3 days to 6 months after treatment, only three had normal stool without *T. foetus* isolated from their stool. Forty percent of the cats in the study continued to have diarrhea after treatment, of which 9 had positive fecal smears, and in the 12 cats that had normal stools, 22% of those cats tested (*n* = 9) had positive fecal smears. Three of the cats in the study were kittens and 2 of them suffered side-effects including acute renal failure, deafness and cataracts. These kittens were included in a case series of 4 kittens that suffered severe side-effects (acute renal failure, deafness and cataracts) after treatment with oral paromomycin [73]. Paromomycin has also been tested in vitro using a 24-h sensitivity assay and the results showed no effect at minimum lethal concentrations (MLC) ≤ 80 μg/mL [74]. Eleven of the original 25 cats were subsequently treated with fenbendazole and then 2-weeks later by furazolidone [3]. There was a decrease in the number of cats having diarrhea, although the stool was described as semi-formed in 9 of the cats with confirmed shedding trichomonads for the following 10-months. Furazolidone has shown equal 24-hour susceptibilities of *T. foetus* cultures to that of metronidazole and ronidazole at MLC of 0.625 to 2.5 μg/mL [74]. In experimentally infected cats, nitazoxanide, a nitrothiazole benzamide compound, with broad-spectrum antiprotozoal activity, decreased the number of trichomonads shed in the feces but failed to eliminate the *T. foetus* infection, although it did eliminate the cryptosporidial infection in those cats that were co-infected. Reported side-effects of nausea and foul-smelling dark diarrhea make it even less attractive as a therapeutic.

Nitroimidazoles (metronidazole, tinidazole and ronidazole) have been investigated both in vitro and in vivo in experimentally induced infections [74-76]. Metronidazole did not show any inhibitory effect in vitro at concentrations ≤10 μg/mL in contrast to tinidazole and ronidazole which had an inhibitory effect at MLC of ≥0.1 μg/mL [75]. Kather et al. demonstrated in vitro results that contradict these findings, as both metronidazole (1.25 to 2.5 μg/mL) and ronidazole (0.625 to 2.5 μg/mL) had a 24-h kill effect on *T. foetus* isolates, although ronidazole had lower 24-h MLC for some isolates [74]. Time-kill assays, which evaluate the degree of growth inhibition and survival during 24-h incubation demonstrated a significant difference in the survival kinetics of *T. foetus* in culture incubated with metronidazole as compared to ronidazole, with ronidazole being more effective [74]. Tinidazole has been investigated twice in vitro with reported MLC of ≥0.1 μg/mL and ≥10 μg/mL [75,76]. The disparity may be ascribable to different strains used in the two experiments.

The efficacy of metronidazole in vitro has not been translated to an in vivo efficacy, although transient improvement in clinical signs do occur independent of elimination of the *T. foetus* infection, the reasons proposed include alteration in bacterial microflora, elimination of *Giardia* co-infection or immunomodulatory effect [33,74]. Interestingly, treatment of infected cats with antibiotics that kill natural microbiota will increase shedding of *T. foetus*, as the organism is dependent on host bacteria for acquisition of micronutrients [75]. Tinidazole cleared experimentally induced *T. foetus* infection within 3-days, but the trichomonads were detected within 6 to 8 weeks after discontinuation of the treatment [76]. The poor in vivo efficacy may be related to its increased absorption and reduced concentration within the intestines. Ronidazole has been investigated in experimentally infected cats and shedding discontinued within 3-days of initiating therapy at 10 mg/kg, dosed orally every 12 h, although relapse was detected but the cultured organisms retrieved from the feces remained susceptible to ronidazole in vitro [75]. Relapses were reported in cats receiving a dose of 30 mg/kg twice daily but not in cats receiving 50 mg/kg twice daily. One reason for better efficacy of ronidazole as compared to other nitroimidazoles may be due to improved trapping of the activated compounds within the intestine [75].

Nitroimidazole resistant trichomonads are hydrogenosomal pyruvate:ferredoxin oxidoreductase deficient, and compensate by increased glycolysis and alternative cytosolic pathways. Ronidazole resistance is defined as aerobic MLC ≥100 μg/mL and is both inherent and acquired [77]. Resistant strains of *T. foetus* to ronidazole have been documented in cats with confirmed treatment failure after excluding the possibility of reinfection or urogenital nidus, and is related to the cross resistance of the organism to all nitroimidazole drugs [77].

Ronidazole is not registered for human or veterinary use and informed consent is necessary prior to use in cats and should only be prescribed in confirmed cases. The current recommended dose is 30 mg/kg, once daily for 14-days [75]. Relapse is common and cats with resolved clinical signs can continue to carry the organism and thus vigilant and prolonged post-treatment monitoring is indicated. No adverse effects were reported in the original study that examined ronidazole as a treatment for *T. foetus* at doses as high as 50 mg/kg, twice daily and hematology and biochemistry results remained within reference range and did not change as compared to pretreatment values [75]. Subsequent to this, four cases of neurological toxicity in cats treated with ronidazole in the range of 30 to 50 mg/kg were reported. The clinical signs included, having a blank stare, “slow motion” movements, agitation, facial tremors, trembling of the extremities, unable to jump or walk stairs and hyperesthesia which started three to nine days after initiating ronidazole, lasting one to four weeks in duration [78]. A lower dose of ronidazole, 10-30 mg/kg once daily, for 14 days, together with a probiotic (Pro-Kolin Enterogenic, Protexin, Probiotics International) was compared to a placebo control and ronidazole at the same dose, frequency and duration in age matched cats diagnosed with *T. foetus*-associated diarrhea [79]. No side-effects were noted with relapse reported in both groups but cats that received the probiotic with the ronidazole were less likely to relapse.

## 11. Conclusions

Feline trichomonosis is caused by *T. foetus*. The etiology was determined merely one and half a decades ago. The current manuscript comprehensively reviews the trichomonad biology and the disease itself. The authors hope this will provides individuals who are interesting in the topic with one source for updated information.

## 12. Abbreviations

bp: base pair
BL: bovine lymphosarcoma
BVEC: Bovine vaginal epithelial cells
CISH: Chromogenic in situ hybridization
CP: Cysteine protease
CVM: College of Veterinary Medicine
dpi: days post infection
EL: Elongation factor
FISH: Fluorescent antibody in situ hybridization
GI: Gastrointestinal
IPEC: Porcine intestinal epithelial cell
ITS: Internal transcribed spacer
LPG: Lipophosphoglycan
MDH: malate dehydrogenase
MDM: Modified Diamond’s Medium
MLC: Minimum lethal concentration
NC: North Carolina
OR: Odds ratio
RAPD: Random amplified polymorphic DNA
RFLP: Restriction fragment length polymorphism
RUSVM: Ross University School of Veterinary Medicine
